# Training and competency frameworks used in the preparation of healthcare professionals for head and neck cancer screening: A scoping review protocol

**DOI:** 10.1371/journal.pone.0330664

**Published:** 2025-09-03

**Authors:** Louise C. Occomore-Kent, Charitini Stavropoulou, Elizabeth C. Ward, Chris Elkington, John C. Hardman, Joanne M. Patterson, Nikki Rousseau, Vinidh Paleri, Madeline Cruice

**Affiliations:** 1 School of Health and Medical Sciences, City St George’s, University of London, London, United Kingdom; 2 The Royal Marsden NHS Foundation Trust, London, United Kingdom; 3 School of Health and Rehabilitation Sciences, The University of Queensland, Brisbane, Queensland Australia; 4 Centre for Functioning and Health Research, Metro South Health, Brisbane, Queensland, Australia; 5 Head and Neck Cancer United Kingdom, Bury St Edmunds, United Kingdom; 6 Liverpool Head and Neck Centre, The University of Liverpool, Liverpool, United Kingdom; 7 Leeds Institute of Clinical Trials Research, University of Leeds, Leeds, United Kingdom; Faculdade Ciencias Medicas de Minas Gerais, BRAZIL

## Abstract

**Objectives:**

This scoping review aims to identify existing training and competency frameworks for healthcare professionals who assess or screen patients on the suspected Head and Neck Cancer (HNC) pathway or in extended practice models, and to explore how skills are developed, and competence assessed, prior to practice.

**Introduction:**

Up to 97% of people referred with suspected HNC in the UK do not have cancer. This contributes to long wait lists and referral to treatment time in HNC services. A potential solution is for Speech and Language Therapists (SLTs) to assist such services by screening low-risk patients, specifically those presenting with hoarseness or oral-pharyngeal dysphagia. If SLTs are to screen low-risk patients safely and effectively, then specific training and competencies are required.

**Inclusion criteria:**

International sources written in English between 2014–2025 that involve training and competencies for healthcare professionals who assess or screen patients for HNC.

**Methods:**

The protocol presents the scoping review process that will be undertaken, which will be conducted as per the Joanna Briggs Institute *Manual for Evidence Synthesis* and reported in accordance with *PRISMA-ScR* guidance. Five databases and grey literature will be searched using a peer-reviewed search strategy. Professional organisations will be contacted for unpublished tools from clinical practice. Titles and abstracts will be screened using an *a priori* protocol. Eligible sources will be charted using an *a priori* framework and will undergo deductive content analysis. Results will be reported quantitatively and qualitatively using flow charts, tables and visual representations to outline the process and present the results.

## Introduction

Over 275,000 referrals are made to the suspected Head and Neck Cancer (HNC) pathway annually in the United Kingdom (UK) [1]. Patients are usually referred by their General Practitioner (GP) for Head and Neck Surgeon-led assessment. UK national targets are to diagnose cancer within 28 days and commence treatment within 62 days of referral [2]. The pathway is commonly still referred to as *the two-week-wait* (2WW) based on prior guidance that patients should receive their first point of contact within 2 weeks of referral. With Ear Nose and Throat (ENT) waiting lists having doubled since the SARS-CoV-2 pandemic, HNC has among the longest wait for treatment of all cancers, with almost half of patients failing to commence treatment within the recommended 62-day standard in the UK [3]. ENT-UK estimates that weekly ENT outpatient clinic and theatre activity would need to double for 1 year to return to pre-pandemic waiting list levels which is unrealistic, particularly considering ENT workforce shortages [4,5].

A potential solution to address these challenges is to utilise the allied health workforce, specifically Speech and Language Therapists (SLTs), to be the first point of contact for a proportion of patients awaiting screening – specifically those classified as low risk, who present with hoarseness or oral-pharyngeal dysphagia as their primary symptom. SLTs specialise in providing advice and treatment for functional voice and swallowing problems, and some already conduct relevant procedures such as flexible nasendoscopy for evaluation of the larynx and swallowing. SLT-led voice clinics for routine ENT referrals have been commonplace in the UK for many years [6]. More recently, in Australia, SLT-led clinics have shown many benefits of utilising SLTs as the first point of contact for low-risk patients referred on the routine ENT pathway including reduced waiting lists, reduced number of patients needing to see ENT, and no adverse events [7], as well as cost efficiency [8]. These findings suggest that with reliable means of identifying low-risk patients on the suspected HNC pathway, similar benefits may be possible in the UK.

Since the deployment of a risk calculator tool during the SARS-CoV-2 pandemic [9], many UK cancer centres now triage their HNC referrals to stratify patients into high- and low-risk categories to prioritise high-risk patients for urgent assessment. The National Institute for Health Research (NIHR)- funded *EVEREST-HN* research programme is currently underway to refine and develop this tool further as a patient-facing risk stratification system to expedite high-risk patients with suspected HNC to appropriate diagnostics and care [10]. This risk stratification advancement may also facilitate identification of low-risk patients appropriate for SLT-led care, as well as directing high-risk patients to HNC surgeons. Two pilot SLT-led clinics for low-risk HNC referrals have shown positive clinical outcomes [11,12] with positive patient experience and no adverse events. Some HNC centres have adopted an alternative nurse-led clinic model [13,14].

Although a SLT model supporting the screening of the low risk suspected HNC patients could be highly beneficial, stakeholder consultation research with both ENTs [15] and SLTs [16] has corroborated that clear training and competencies will be required to prepare SLTs undertaking this type of role with endorsement from relevant professional, statutory and regulatory bodies of both professions. Such training and competencies would need to meet the needs of a variety of different services due to variation in local healthcare settings and needs [17]. Yet, at present there is limited knowledge about what is needed to prepare the SLT for this type of role. Hence there is a need to understand what training is undertaken by SLTs in similar extended scope roles such as those abroad, and other health professionals providing screening services for HNC to determine what of this could be used in SLT role preparation. There is no current, complete competency framework for healthcare professionals to undertake this extended scope role to the authors’ knowledge. It is our aim that such a competency framework may be informed, in part, by the current scoping review.

### Aims

The primary aim of the scoping review will be to identify existing training and competency frameworks used for healthcare professionals who already assess patients on the suspected HNC pathway or who screen for HNC in other relevant extended scope pathways. A competency framework is defined by the World Health Organisation [18] as ‘an organized and structured representation of a set of interrelated and purposeful competencies’, with competencies defined as ‘the abilities of a person to integrate knowledge, skills and attitudes in their performance of tasks in a given context’. Professionals in the current scoping review may include ENT surgeons, nurses, and Physician Associates among others. Training frameworks may include documents that detail education and training programmes to prepare a healthcare professional to undertake the role, e.g., tools that detail the content of such training and/or the pedagogy of how these are developed. Competency frameworks may constitute tools that outline what a healthcare professional needs to know or be competent to perform and/ or the method by which this is assessed. Training and competencies may be within one document or may constitute separate sources and tools.

This scoping review aims to identify existing training and competency frameworks used to train healthcare professionals who already assess patients on the suspected HNC pathway or in other extended scope clinic models in ENT. It aims to elucidate how these are skills are developed and any competencies assessed prior to practice. This will inform future development of a training and competency framework for SLTs in this type of role.

### Research question

Developed using a Population, Concept, Context (PCC) [19] format, the research question is as follows:

What is the nature of post-registration training and competence (concept) of healthcare practitioners (population) involved in screening patients for head and neck cancer (context)?

### Rationale

No prior scoping or systematic reviews, or protocols, have been published about this specific topic.

The extracted data will produce a list of training or competency requirements for healthcare professionals assessing patients on the suspected HNC pathway, based on the current sources available. For example, a requirement might be to be able to carry out a neck examination independently. The method by which these are developed and how competence is measured will also be included. For example, this may be for a head and neck surgeon to observe the developing SLT completing a neck examination independently on 10 occasions to a satisfactory standard. It is anticipated that there will be variability in training and competency approach by profession, region and setting. These data will therefore be used to inform an e-Delphi survey of expert ENT and SLT clinicians who will be asked to agree which of these items are important and necessary for a training and competency protocol specifically for the SLT profession. The resulting framework will then be piloted with healthcare professionals to assess its feasibility, effectiveness and acceptability.

A scoping review methodology will be utilised as many healthcare policies, guidelines, frameworks and competencies appear in grey literature, e.g., professional and statutory body guidelines. Appraisal of data quality is not an aim of this review consistent with scoping review methodology [19]. However, credibility of sources will be assessed with reference to CRAAP [20] criteria as well as exploring whether they are theoretically or empirically informed. Exploration of the breadth and methods of any available tools is the rationale. A scoping review to inform the future e-Delphi consensus work is therefore the most appropriate evidence synthesis method.

The objective of the scoping review will be to identify all current training and competency tools and frameworks used to develop and measure competencies for healthcare professionals who already assess patients referred on the suspected HNC pathway or who screen for HNC as part of other extended scope of practice clinic models. The process by which these competencies are developed, and their measurement, will also be explored.

## Method

The methodology for the review will be guided by the Joanna Briggs Institute *Manual for Evidence Synthesis [*19*]* and will be reported with reference to *PRISMA-ScR* guidelines [21]. A *PRISMA-ScR* checklist has informed the current protocol (S1 File).

### Inclusion criteria

#### Population.

Articles and documents intended for any and all healthcare professionals will be included in this scoping review. This includes, but is not exclusive to, ENT surgery trainees, Nurses, SLTs, Physician Associates, and GPs with Extended Role in ENT.

#### Concept.

All and any tools containing specific items that a healthcare professional needs to know or be competent to perform when screening first point of contact ENT patients including, but not limited to, competency documents, knowledge and skills frameworks and training protocols.

#### Context.

Articles and documents will be included where they relate to the suspected HNC pathway (formerly the 2WW pathway) or to related extended scope of practice models such as SLT- or nurse-led clinics in first point of contact ENT clinics.

### Types of evidence sources

All sources of information will be included such as peer reviewed journal articles, clinical guidelines, clinical policies, competency frameworks and training protocols among others.

### Search strategy

#### Stage 1.

A pilot search will be conducted in 2 online databases: MEDLINE Complete (EBSCOhost) and AMED (Ovid Online).

#### Stage 2.

Index terms and text words in the titles and abstracts of the retrieved sources will be used to inform further searches in all databases. These will include Embase (Ovid Online), CINAHL (EBSCOhost), Web of Science, Scopus and Google Scholar.

The same index terms and key text words will inform a search of grey literature databases and search engines including Overton, Trip and Google. Relevant Professional, Statutory and Regulatory Bodies (PSRBs) in the United Kingdom (UK) will be contacted directly for any relevant training or competency tools currently in development including British Association of Head and Neck Oncologists (BAHNO), Royal College of General Practitioners (RCGP), and Royal College of Surgeons (RCS) among others. Clinical and research specialist networks will be contacted for the same information. For example, the Clinical Excellence Networks for HNC and the National Institute for Health Research’s (NIHR) research group for SLTs with special interest in ENT (STENT). The current scoping review is being conducted to inform practice in the UK specifically and only UK-based organisations have been included to keep appraised of any tools in development specific to that region. Corresponding authors will be contacted directly for more information where required. For example, if a source states that training was provided for healthcare professionals when piloting or implementing a new clinic without details of what this entailed, the detail will be requested. Contact will be made by email initially, with a further email a fortnight later if no response is received. The number of authors contacted, and their responses will be recorded and reported.

Stakeholder involvement will be reported with reference to the ACTIVE framework [22] Stakeholders will include *knowledge users* [23] a Patient and Public Involvement and Engagement (PPIE) Group comprising 4 patients with experience of the suspected HNC pathway and a Clinical Advisory Group (CAG)- comprising a HNC surgeon and three expert SLTs with experience in developing and delivering training frameworks for SLT extended scope of practice and working in the HNC pathway. Members of both groups have been recruited by invitation. Involvement will be continuous and stakeholders in the PPIE group will be remunerated for their time. Both the PPIE group and the CAG have been involved prior to the start of the scoping review, with one PPIE member being a co-author on the current protocol (CE) and attending monthly project team meetings. Involvement will be continuous with both groups via direct interaction. Both groups will contribute to and influence the scoping review. Author CE will co-lead the PPIE meetings. Our PPIE members will also be encouraged to lead on some dissemination activities following the review if they wish. PPIE training will be offered to group members if required, and PPIE members will be remunerated for this training time. Items extracted from this scoping review will inform a 3-stage e-Delphi study in future with expert clinicians. This is beyond the period of the current scoping review and will be reported separately.

#### Stage 3.

The reference lists for articles selected and included for full text analysis will be examined for any other relevant papers. The titles and abstracts of those sources will be screened, and full texts will be obtained as appropriate.

International sources will be searched. Only those published in English will be included due to resource constraints. The time frame to be examined is 2014–2025. Extended scope roles for AHPs have only become commonplace in the past decade, with very limited literature on the topic prior to this [24]. Additionally, any guidelines, training protocols or competency frameworks developed prior to 2014 are unlikely to be relevant to current clinical practice or would have been updated more recently if still in use. The *Multi-Professional Framework for Advanced Clinical Practice in England* was published in 2017 [25] which, along with the *AHPs into Action* guidance [26], expedited extended and advanced clinical practice roles in the UK for AHPs. The 2014–2025 time frame was agreed by consensus with the CAG on this basis.

Sources will be uploaded to the reference management software Refworks (ProQuest) and will be combined and de-duplicated in Refworks prior to screening. Screening will take place in the reviewer software Rayyan [27].

The full search strategy will be added as an appendix to the main scoping review article. A preliminary search strategy has been developed in MEDLINE Complete (EBSCOhost) and peer reviewed by a subject librarian (Table 1). The scoping review will commence following this protocol’s development and any deviation will be reported in the full scoping review manuscript.

**Table 1:** Search strategy for Medline Complete (EBSCOhost).

Search	Search Terms	Search modes
S1	(MH “Health Personnel”) OR (MH “Physical Therapists”) OR (MH “Health Educators”) OR (MH “Physicians”) OR (MH “Surgeons”) OR (MH “General Practitioners”) OR (MH “Oral and Maxillofacial Surgeons”) OR (MH “Otolaryngologists”) OR (MH “Oncologists”) OR (MH “Personnel, Hospital”) OR (MH “Medical Staff, Hospital”) OR (MH “Nursing Staff”) OR (MH “Nursing Staff, Hospital”) OR (MH “Nurses”) OR (MH “Nurse Practitioners”) OR (MH “Nurse Specialists”) OR (MH “Nurse Clinicians”) OR (MH “Medical Staff”) OR (MH “Allied Health Personnel”) OR (MH “Physician Assistants”) OR (MH “Educational Personnel”)	Proximity
S2	(MH “Speech-Language Pathology”)	Proximity
S3	AB (“health professionals” or “health personnel” or “healthcare provider” or “allied health” or otolaryngologists or surgeons or nurses or “nurse practitioner” or “speech and language therap*” or “SLT” or ENT)	Proximity
S4	S1 OR S2 OR S3.	Proximity
S5	(MH “Teaching”)	Proximity
S6	(MH “Education”)	Proximity
S7	(MH “Clinical Protocols”)	Proximity
S8	(MH “Clinical Competence”) OR (MH “Professional Competence”)	Proximity
S9	(MH “Aptitude”)	Proximity
S10	AB (teaching or training or education or protocols or competencies or framework or skills)	Proximity
S11	S5 OR S6 OR S7 OR S8 OR S9 OR S10.	Proximity
S12	(MH “Head and Neck Neoplasms”) OR (MH “Otorhinolaryngologic Neoplasms”) OR (MH “Pharyngeal Neoplasms”)	Proximity
S13	(MH “Otolaryngology”)	Proximity
S14	(MH “Otorhinolaryngologic Diseases”) OR (MH “Laryngeal Diseases”) OR (MH “Voice Disorders”) OR (MH “Dysphonia”) OR (MH “Hoarseness”) OR (MH “Aphonia”) OR (MH “Laryngeal Neoplasms”) OR (MH “Laryngeal Edema”) OR (MH “Granuloma, Laryngeal”) OR (MH “Vocal Cord Paralysis”) OR (MH “Vocal Cord Dysfunction”) OR (MH “Laryngitis”)	Proximity
S15	(MH “Outpatient Clinics, Hospital”)	Proximity
S16	AB (“head and neck cancer” or “oral cancer” or “oropharyngeal cancer” or hnc or “2ww” or “two week wait” or “two-week-wait” or “urgent ENT”)	Proximity
S17	S12 OR S13 OR S14 OR S15 OR S16.	Proximity
S18	S4 AND S11 AND S17.	Proximity

### Scoping review timeline

The draft of this protocol was prepared in January 2025, prior to the commencement of the scoping review and was submitted to the journal on 18^th^ March 2025 following approval from all co-authors. The pilot database search in MEDLINE Complete using the search terms in Table 1 was conducted on 7^th^ February 2025 to further refine the search in preparation for the commencement of the main review. The main scoping review search strategy will commence after this protocol is submitted on 19^th^ March 2025. The results are expected by June 2025 and will be prepared for publication after this date.

### Evidence selection

Following deduplication, 2 reviewers will independently screen 100 titles and abstracts (these have been randomly sampled) and a minimum of 75% inter-rater reliability is required [28]. Disagreements will be resolved through firstly discussion between the two reviewers to seek consensus; where consensus cannot be achieved disagreements will be brought for third party adjudication. Screening criteria for inclusion will relate to the PCC question whereby sources must relate to the population, concept and/ or context. Sources will be excluded where these are irrelevant to the PCC question, for example if the training relates to surgical techniques, an unrelated population, e.g., children, or an irrelevant context, e.g., the emergency room. Where an article’s eligibility is unclear from the title and abstract, the full text will be retrieved, for example where a source details implementing a new extended scope clinic in ENT without specifically mentioning training in the title or abstract, but this may conceivably be part of the implementation process in the full text. Articles deemed to be eligible based on title and abstract screening will progress to full text review. Two reviewers will independently review all full text sources. Disagreements will be considered between reviewers to seek consensus. Where consensus cannot be achieved, third party adjudication will be employed.

A flowchart will be provided detailing each stage of the review and the full text articles retrieved. Excluded sources at the full text review stage will be detailed in an appendix with brief rationale for their exclusion.

### Data extraction and synthesis

Data will be extracted and charted using Table 2. The chart has been piloted using 3 sources but may be adapted further during the full review as scoping reviews may be iterative in their approach. The reviewers will meet frequently throughout the period of data extraction to discuss queries and conflicts and to adapt the approach iteratively. Any adaptations made to the chart will be outlined in the main scoping review manuscript with rationale.

**Table 2:** Data extraction chart.

Source	Type	Credibility	Healthcare profession	Sample characteristics	Within/ advanced scope	Skill	Training method	Duration/ frequency	Competency measurement	Profession/ experience of trainer	Skill maintenance	Evaluation details
*e.g., GMC website*	*e.g., PSRB guideline*	*e.g., C.R.A.A.P. [* 20 *] rating, peer review and theoretical base*	*e.g., ENT resident*	*e.g., training provided to 5 female ENTs and 8 male ENTs across 2 London hospitals. Mean qualification duration ~3 years. Ethnicity and age not reported.*	*e.g., within scope*	*e.g., neck exam*	*e.g., simulation and supervised practice*	*e.g.,* *watch 3*	*e.g., supervisor signs off after 3 competent examinations of a patient’s neck*	*e.g., must be Consultant HNC surgeon*	*e.g., must complete at least 10 exams per year to remain competent*	*e.g., training process was evaluated with 10 post-registration nurses. Nurses self-evaluated own competence and competence pre and post training with x outcome.*

### Data analysis

The type of data to be charted in this scoping review will be both qualitative in nature, i.e., items of knowledge and particular skills that a healthcare professional requires when screening for HNC, and quantitative, e.g., the need to carry out a procedure *x* number of times to be deemed competent. Content analysis will be applied to analyse the data, which are anticipated to be largely qualitative in nature. As the scoping review’s research question aims to identify characteristics of a concept, synthesis approaches such as meta-analysis are not required and are inappropriate in a scoping review methodology [19].

A 3-stage content analysis approach will be applied as described by Elo and Kyngäs [29].

1. *Preparation Phase*: A deductive approach will be taken to the content analysis using an *a priori* framework related to the research questions (Table 3).
2. *Organising stage*: 2 researchers will extract verbatim information from each source and add it to the framework. The researchers will meet frequently during this process to discuss the process and if a more inductive approach is required then the approach may be adapted, then outlined and justified in the final scoping review manuscript.
3. *Reporting stage*: PRISMA-ScR guidelines [21] will inform the reporting of the scoping review findings, and a checklist will be included with the final manuscript.

**Table 3:** A-priori framework for content analysis.

Skill or knowledge item required for role	Method of teaching or training	Method of measuring achievement/ competence
*e.g., to conduct neck exam to identify any palpable neck masses*	*e.g., to observe 10 by ENT, to conduct 50 under ENT supervision*	*e.g., ENT to sign off competency log with sections on explaining procedure to the patient, conducting the exam thoroughly, and explaining the findings to the patient.*

### Presentation of results

The results will be presented in a way that communicates the findings to knowledge users engagingly which may include tables, graphs, and/ or visual representations, taking into account the volume of data and how the findings can be most effectively communicated through publication. We will suggest directions for future research as appropriate to the scoping review’s findings.

## Supporting information

S1 File Preferred Reporting Items for Systematic reviews and Meta-Analyses extension for Scoping Reviews (PRISMA-ScR) Checklist. (DOCX)
